# Risk Factors Associated With Secondary Measles Transmission in Index Cases

**DOI:** 10.2188/jea.JE20250293

**Published:** 2026-10-05

**Authors:** Yuki Takahashi, Hideo Tanaka, Toshiyuki Shibata, Chisato Tanikake, Takashi Moriwaki, Masaya Miyazono, Sayuri Matsumoto, Masaru Kinoshita, Hitomi Nagai, Chika Shirai, Yoshihiro Takayama, Megumi Okada, Yuko Tsuda, Ayako Hazama, Toshitake Ohara, Hideki Yoshida, Takako Kurata, Daiki Kanbayashi, Chie Taniguchi, Kazushi Motomura

**Affiliations:** 1 Health and Medical General Affairs Division, Osaka Prefectural Government, Osaka, Japan; 2 Moriguchi Public Health Center of Osaka Prefectural Government, Osaka, Japan; 3 Takatsuki City Public Health Center, Osaka, Japan; 4 Izumisano Public Health Center, Osaka Prefectural Government, Osaka, Japan; 5 Kishiwada Public Health Center, Osaka Prefectural Government, Osaka, Japan; 6 Higashi-Osaka City Health Center, Osaka, Japan; 7 Ibaraki Public Health Center, Osaka Prefectural Government, Osaka, Japan; 8 Hirakata City Public Health Center, Osaka, Japan; 9 Yao City Public Health Center, Osaka, Japan; 10 Osaka City Public Health Office, Osaka, Japan; 11 Fujiidera Public Health Center, Osaka Prefectural Government, Osaka, Japan; 12 Izumi Public Health Center of Osaka Prefectural Government, Osaka, Japan; 13 Osaka City Public Health Bureau, Osaka, Japan; 14 Osaka Institute of Public Health, Osaka, Japan; 15 College of Nursing, Aichi Medical University, Aichi, Japan

**Keywords:** measles, vaccination, secondary transmission

## Abstract

**Background:**

Limited information has been shown quantitatively assessed factors associated with the risk of secondary transmission in measles index cases.

**Methods:**

We collected data on measles index cases reported in Osaka Prefecture between November 2018 and March 2019, along with their close contacts. Secondary attack rates (SARs) in contacts of index cases were calculated and compared depending on the vaccination status, classical or modified measles and other risk factors in index cases. For the multivariate analyses, we used binary logistic regression to adjust for these potential confounding factors on the secondary transmission and calculated the adjusted odds ratio (aOR) for the SARs.

**Results:**

A total of 105 index cases were included, involving 9,846 close contacts, with 72 secondary cases identified. A significant negative association between the number of vaccinations received by index cases and the risk of secondary infection in his/her close contacts was observed (*P* < 0.01). Index cases who had received one vaccine dose had an aOR of 0.22 (95% confidence interval [CI], 0.08–0.65), while those with two or more doses had an aOR of 0.25 (95% CI, 0.07–0.85). Index cases who did not exhibit all three classic measles symptoms had a significantly lower risk of causing secondary infections compared to those who presented with all three symptoms (aOR 0.12; 95% CI, 0.03–0.39).

**Conclusion:**

The indirect protective effect of measles vaccination would present important evidence supporting the further strengthening of vaccination policies.

## INTRODUCTION

Measles is caused by the *Morbillivirus hominis* virus, a member of the genus Morbillivirus within the family Paramyxoviridae.^1^ Each of the three classic clinical symptoms—rash, fever, and catarrhal signs—occurs in approximately 90% in non-vaccinated individuals, whereas their frequency is substantially lower among vaccinated cases.^2^^,^^3^ Children are especially at risk of severe complications, including pneumonia and encephalitis, highlighting the need for measles to remain a major focus of global public health efforts.

The number of reported measles cases worldwide declined along with the increased coverage of the measles vaccine from 853,000 in 2000^4^ to 123,000 in 2021.^5^ However, large-scale resurgences were observed in both 2019 and 2023, with each year reporting over 600,000 cases, indicating that the threat of widespread measles outbreaks still remains.^4^

Measles-related deaths were reported annually at least until the year 2000 in Japan, and the disease was widely regarded as a serious public health concern, prompting vaccination policies.^6^ Japan implemented a routine measles vaccination program (single-dose) for infants using a measles-only vaccine in 1978.^7^ The measles, mumps, and rubella combination vaccine was introduced in 1989, but it was discontinued in 1993 due to adverse reactions, such as aseptic meningitis. A two-dose schedule using the combined measles-rubella (MR) vaccine was introduced in 2006. Children received routine vaccinations at ages 1 and 5–6 years. Therefore, individuals who were between 5–6 and 18 years old in 2019 were covered by the two-dose routine vaccination schedule. Additional vaccination programs were instigated in an effort to improve antibody prevalence among adolescents between 2008 and 2012. These efforts mainly targeted 12- to 18-year-olds who had not received the two-dose MR vaccination schedule, administering additional doses to this age group. Vaccination coverage in Japan has consistently been around 95% for the first dose and above 90% for the second dose each year since the introduction of the two-dose schedule, at least after 2010.^5^ As a result of these vaccination measures, the measles antibody prevalence (particle agglutination method, antibody titer of 1:16 or higher) in Japan, including Osaka Prefecture, reached over 95% for all age groups 2 years and older by 2019.^8^^,^^9^

Japanese government officially declared the elimination of measles in 2015,^10^ after strengthened vaccination policies proved effective. Despite this, several hundred cases were reported annually from 2016 to 2019, primarily due to outbreaks triggered by imported cases.^11^ Although annual measles cases were below 10 during the coronavirus disease 2019 pandemic, probably due to decrease in international travel, it increased to over 20 cases after 2023 in Japan. From early 2018 through mid-November 2018 in Osaka Prefecture, the number of weekly reported measles cases was very low, occasionally a single case.^12^ Then the outbreak started in late November 2018 in Osaka, with weekly occurrences frequently around 4 cases. From January to March 2019, weekly reports frequently exceeded 10 cases, with the numbers peaking at 28 cases in the week of February 11–17. After the peak, this local endemic wave was largely under control by the end of March 2019, although the official end of this local endemic was May 2019.

Public health authorities in some measles eliminated countries, including Japan, conduct an epidemiological investigation that involves identifying close contacts, administering emergency vaccinations, and monitoring their health status.^13^ To effectively prevent the spread of infection, it is ideal to assess the infectiousness of each measles index case and adjust the identification of close contacts based on the level of risk. Descriptive epidemiological studies have documented that vaccinated measles cases generally exhibit lower infectiousness compared to unvaccinated cases.^14^^,^^15^ However, few studies have applied analytical epidemiological methods to quantitatively estimate the extent to which vaccination reduces the risk of secondary transmission. Furthermore, there is limited research on other factors that may influence infectiousness. The present study therefore utilized data from measles cases and their close contacts reported in Osaka Prefecture (population 8.8 million in 2015) during 2018–2019 to quantitatively evaluate the index case factors associated with the risk of secondary transmission in a retrospective design.

## METHODS

### Subjects

The subjects of this study were primary measles cases (index cases) reported in Osaka Prefecture between November 2018 and March 2019 and their close contacts. Contacts were excluded if an epidemiological link with domestic measles cases other than their respective primary case was identified through epidemiological investigations conducted by public health centers.

Measles cases were defined as individuals presenting with symptoms suggestive of measles, who were subsequently diagnosed through polymerase chain reaction (PCR) testing or immunoglobulin M (IgM) antibody testing. We defined classical measles who showed all three clinical symptoms of 1) generalized rash; 2) high grade fever >38.5°C; and 3) cough, coryza, and conjunctivitis. Modified measles was defined as cases presenting with one or two of the three classical measles symptoms.

Laboratory testing for measles is generally conducted in the following cases in Osaka: 1) when the patient presents with all three classic symptoms; and 2) when one or two of these symptoms are present, and there is a confirmed history of contact with a measles case. However, in some instances, testing may be performed at the discretion of the attending physician, even if these criteria are not fully met.

The genotype was determined on the 450-nucleotide region of MV that codes for nucleoprotein N-450 (positions 1233–1682) following World Health Organization recommendation.^16^

### Data collection items

When physicians suspect measles in a patient, they conduct IgM testing (4 to 28 days after symptom onset) and PCR testing where possible in Japan. Clinical information of measles is then reported to the local public health center, as required by the Infection Control Law. The public health center then approached the measles case to identify his/her close contacts based on the patient’s travel and contact history. To ensure accurate information, public health nurses ask patients or their family members by telephone to check vaccination records in mother-and-child health handbooks or official vaccination certificates issued after vaccination and report the details. If the documents cannot be found, the information is obtained based on the recollection of the patient or their parents. The identified contacts are monitored for their health status for 21 days from the last contact with their index cases. If any of them exhibit symptoms suggestive of measles, the public health center advises them to seek medical attention, and the results of their examination are subsequently tracked.

We collected the following information based on reports from physicians and interviews conducted by the public health center:

#### Information on index cases

• Basic information (sex, age, vaccination history)
• Three classic symptoms (fever, rash and catarrhal symptoms (cough, coryza and conjunctivitis)).
• Laboratory tests (eg, results of IgM and PCR testing)

#### Information on close contacts of each index case

• The number of close contacts
• The number of secondary cases

A close contact was defined as anyone who had direct contact with a measles patient during the infectious period (from 1 day before the onset of measles symptoms to 3 days after the fever subsides), anyone who may have inhaled droplets from the patient’s cough, sneeze, or conversation within a 2-meter radius, or anyone who shared an enclosed space (including spaces with air conditioning) with the index cases.^17^ Secondary cases were defined as close contacts of index cases who developed symptoms consistent with measles and were laboratory-confirmed by PCR or IgM antibody testing.

### Analysis

Secondary attack rates (SARs) were calculated as the number of secondary cases divided by the total number of close contacts of each index case. SARs were then compared depending on sex, age, number of contacts, vaccination status, symptoms and IgM test result in index cases. Index cases were categorized as ≤14, 15–24, 25–39, or ≥40 years. Number of contacts of each index cases was categorized as 1–9, 10–99, or ≥100 contacts. Symptoms were categorized as classical measles or modified measles. The statistical significance was assessed by chi-square test. For the multivariate analyses, we used binary logistic regression to adjust for these potential confounding factors on secondary transmission and calculated the adjusted odds ratio (aOR) for the SARs. The factors adjusted for included sex, age, number of contacts, vaccination status, and symptoms. Statistical analysis was performed using the R software (ver. 4.4.2; R Foundation for Statistical Computing, Vienna, Austria).

This study was carried out in accordance with the Infectious Diseases Control Law and received approval from the Ethics Committee of the Osaka Institute of Public Health (approval No. 1912-06).

## RESULTS

### SARs according to factors in index cases

We collected 105 index cases in the study period. The total number of close contacts corresponding to these cases was 9,846, with an average of 93.7 close contacts per index case (Table 1). The total SAR in close contacts was 0.73% (72/9,846). Contact with a male index case was associated with a higher SARs compared to those with a female index case (1.14% vs 0.38%, *P* < 0.01). The contacts whose index case received the vaccine more than twice had a lower SAR than those whose index case received no vaccine (0.13% vs 1.19%, *P* < 0.05) and a negative correlation between the number of vaccinations and the risk of secondary infection of his/her contacts (*P* < 0.01). The contacts whose index case had two or fewer of the classic symptoms also had a significantly lower SAR than those whose index case had all three classic symptoms (0.12% vs 0.93%, *P* < 0.01). Close contacts of index cases with a negative IgM test had a lower SAR compared to those with a positive result (0.00% vs 1.24%, *P* < 0.01).

**Table 1.** Secondary attack rates among contacts of index cases with measles, stratified by potential risk factors in index cases

Variables in index cases	Index cases	Contacts	Infected contacts	Secondary attack rate	95% confidence interval	*P*-Value
Sex						*P* < 0.01^a^
Male	52	4,566	52	1.14%	0.83–1.45%	
Female	53	5,280	20	0.38%	0.21–0.54%	

Age, years						
≤14	12	1,479	7	0.47%	0.12–0.82%	*P* < 0.05^b^
15–24	29	1,952	24	1.23%	0.74–1.72%	—
25–39	51	4,387	30	0.68%	0.44–0.93%	*P* < 0.05^b^
≥40	13	2,028	11	0.54%	0.22–0.86%	*P* < 0.05^b^

Number of contacts						*P* < 0.01^c^
1–9	27	105	7	6.67%	1.90–11.44%	
10–99	49	2,069	27	1.30%	0.82–1.79%	
≥100	29	7,672	38	0.50%	0.34–0.65%	

Vaccination						*P* < 0.01^c^
0	54	5,035	60	1.19%	0.89–1.49%	
1	21	2,019	4	0.20%	0.00–0.39%	
≥2	22	2,283	3	0.13%	0.00–0.28%	
Unknown	8	509	5	0.98%	0.13–1.84%	

Symptoms						*P* < 0.01^a^
Classical measles	61	7,414	69	0.93%	0.71–1.15%	
Modified measles	44	2,432	3	0.12%	0.00–0.26%	

IgM						*P* < 0.01^a^
Positive	47	4,922	61	1.24%	0.93–1.55%	
Negative	10	748	0	0.00%	0.00–0.00%	
Unknown	48	4,176	11	0.26%	0.11–0.42%	

Total						
	105	9,846	72	0.73%	0.56–0.90%	

^a^Chi-squared test *P*-value excluding unknown data.

^b^Chi-squared test *P*-value compared with 15–24 years age group.

^c^*P* for trend excluding unknown data.

### Associations between factors in index cases and the risk of secondary infection

Multivariate logistic regression analysis showed a significant negative association between the number of vaccinations received by index cases and the risk of secondary infection in his/her close contacts (*P* < 0.01) (Table 2). Specifically, index cases who had received one vaccine dose had an aOR of 0.22 (95% confidence interval [CI], 0.08–0.65), while those with two or more doses had an aOR of 0.25 (95% CI, 0.07–0.85). Index cases who did not exhibit all three classic measles symptoms had a significantly lower risk of causing secondary infections compared to those who presented with all three symptoms (aOR 0.12; 95% CI, 0.03–0.39). Index cases aged ≤14 years and those aged ≥40 years had significantly lower risk of causing secondary infections than those aged 15–24 years (aOR 0.32; 95% CI, 0.13–0.77 and aOR 0.37; 95% CI, 0.17–0.78, respectively).

**Table 2.** Factors in index cases associated with the risk of secondary measles transmission among their contacts

Variables in index cases	Adjusted odds ratio (95% CI)^a^	*P* for trend^b^
Sex		—
Male	—	
Female	0.42 (0.24–0.73)	

Age, years		—
≤14	0.32 (0.13–0.77)	
15–24	—	
25–39	0.63 (0.36–1.10)	
≥40	0.37 (0.17–0.78)	

Number of contacts		*P* < 0.01
1–9	—	
10–99	0.10 (0.04–0.26)	
≥100	0.04 (0.02–0.09)	

Vaccination		*P* < 0.01
0	—	
1	0.22 (0.08–0.65)	
≥2	0.25 (0.07–0.85)	
Unknown	0.77 (0.29–2.04)	

Symptoms		—
Classical measles	—	
Modified measles	0.12 (0.03–0.39)	

CI, confidence interval.

^a^Adjusted for sex, age, number of contacts, number of vaccine doses and symptoms.

^b^*P* for trend excluding unknown vaccination data.

## DISCUSSION

There is a significantly lower risk of causing secondary infection of close contacts when measles index cases have been vaccinated. Similarly, index cases with modified measles had significantly lower risk of causing secondary infection of their close contacts. Such quantitative assessments of the indirect protective effects of measles vaccination have been rare in previous research.

Several descriptive epidemiological studies have reported that no or very few secondary cases arose when the index cases had been vaccinated.^14^^,^^15^ A systematic review reported that the effective reproduction number among vaccinated measles cases was estimated at 0.063 based on 14 studies and the secondary attack rate ranged from 0% to 6.25% in three studies with clearly identified numbers of close contacts.^18^ Mizumoto et al analyzed data from the 2018 measles outbreak in Okinawa Prefecture (Japan) using mathematical statistical models. They hypothesized that even measles patients with fewer than three classic symptoms (modified measles) could potentially cause secondary transmission, albeit with a low probability.^19^ Gastañaduy et al analyzed measles cases in the United States from 2001 to 2017 and reported that the effective reproduction number was 0.76 among unvaccinated individuals, 0.17 among those who had received one vaccine dose, and 0.27 among those who had received two or more doses, indicating markedly reduced transmissibility in vaccinated cases.^20^ While the above studies suggest that vaccinated index cases pose a markedly lower risk of causing secondary infections, a detailed, quantitative evaluation with reference to the non-vaccinated index cases had not been conducted thus far. In contrast, a major strength of the current analytical epidemiological study is the quantitative evaluation of the reduction in secondary infection risk associated with vaccination.

It has been reported that vaccinated cases shed lower amounts of virus.^18^^,^^21^ This suggests that the suppression of viral replication, likely due to immunological mechanisms, explains the association between vaccinated index cases and the reduced risk of secondary infection. Modified measles with less than the three classic symptoms typically occurs not only in vaccinated measles cases but also in pediatric patients still with maternal antibodies.^22^ It is possible that some degree of immunity is maintained when classical measles symptoms are minimal, explaining the association between fewer symptoms and the risk of secondary infection. Furthermore, several studies have suggested that the intensity of coughing influences the distance of droplet dispersion, which may also contribute to the reduced risk of transmission.^23^ This mechanism on the symptom would reduce the risk of transmission from modified measles patients.

We found that primary cases aged 15–24 years had a higher probability of transmitting the infection to their contacts compared to 0–14 age groups.^11^ Japan implemented two-dose routine measles vaccination, the first dose for 1-year infants and second for children from ≥5 to <7 years in 2006. Individuals aged 18 years and older had a higher rate of receiving only one vaccine dose (≥30%) and a lower rate of two-dose coverage (≤50%) compared to those aged 6–17 years (approximately 20% and 70%, respectively).^24^ This difference is likely due to the introduction of the routine second dose for children aged 5–6 years, which applied to those who were under 18 years in 2019. Primary cases aged 14 years or younger were more likely to have frequent contact with peers who had received two doses, particularly in settings such as schools and daycare centers. This may have contributed to a lower transmission rate in this age group compared to those aged 15–24 years. Index cases among those aged 15–24 years also showed relatively higher infectivity compared to those aged 40 years and older. The annual number of measles cases reported in Japan since 2009 has ranged from 0 to 1,000, whereas prior to 2008, there were years with reports in the tens of thousands.^25^ Older individuals are more likely to have acquired immunity through past infections, which is possibly associated with lower infectivity among peers of the older index cases. To accurately assess the relationship between age and infectivity, it is necessary to confirm the history of vaccination and past infections among both the primary cases and their close contacts.

Close contacts of index cases with a negative IgM test had a lower SAR compared to those with a positive result in this study. Some studies have reported that vaccinated measles cases often do not show detectable IgM levels, likely because vaccination triggers a strong IgG response that suppresses the IgM reaction during subsequent infections.^14^^,^^26^^,^^27^ A negative IgM result may indicate sufficient immunity, which could show a lower risk of infectivity. However, we were unable to collect information on the exact dates when IgM testing was conducted. During the early stages (the first few days), IgM antibodies may not be detectable.^13^ We also need to consider the shorter time from onset to diagnosis allowed for earlier isolation as one contributing factor. Moreover, evaluating the relationship between immune status and infectivity may require additional testing, such as IgG antibody.^13^

Index cases with fewer contacts tended to exhibit higher secondary attack rates (SARs). This may be attributed to a higher proportion of close contacts, such as household members, among their contacts. Such close proximity is speculated to account for the elevated SAR observed in these index cases.

The present study has several limitations. First, the measles vaccination history of the close contacts was not available. Vaccination history is influenced by factors such as place of residence, religion, economic background, and education level.^28^ It is plausible to consider that these characteristics tend to be similar among family and friends of index cases.^20^ Therefore, these factors might overestimate the risk of causing secondary infections from index cases without vaccination history. A second limitation is that the presence of the three classic symptoms was recorded as a single combined variable, and information on each symptom individually was not available. Consequently, we could not assess whether cough was independently associated with the risk of secondary transmission. Future studies should collect data on each of them separately to more precisely evaluate the association between cough and transmissibility. Third, the lack of consideration of environmental factors beyond the measles patients themselves that could influence transmission. In cases where the number of close contacts was large, exposure may have occurred in more spacious environments, such as mass gatherings, where the risk of transmission might have been lower compared to household settings.

The present study indicates that the vaccination history of index cases and the presence of the three classic symptoms of measles (fever, rash, and catarrhal symptoms) are strongly associated with the degree of risk of secondary transmission. The indirect protective effect of measles vaccination would present important evidence supporting the further strengthening of vaccination policies.
